# Daily web survey data collection of time-varying cannabis use motives and contexts, with implications for adaptive interventions: A pilot study

**DOI:** 10.1016/j.drugalcdep.2025.112974

**Published:** 2025-11-21

**Authors:** Yongchao Ma, Brady T. West, Sean Esteban McCabe

**Affiliations:** a Survey Research Center, Institute for Social Research, University of Michigan, Ann Arbor, United States; b Center for the Study of Drugs, Alcohol, Smoking and Health, School of Nursing, University of Michigan, Ann Arbor, United States

**Keywords:** Motives for cannabis use, Contexts for cannabis use, Adaptive interventions, Latent transition analysis, Daily web survey

## Abstract

**Background:**

With the rising prevalence of daily cannabis use in the U.S., more individuals may seek treatment for adverse outcomes (e.g., cannabis use disorder) arising from frequent cannabis use. Adaptive interventions leverage self-reported motives for cannabis use to develop personalized support. Research has demonstrated that these motives can be collected on a yearly, monthly, or daily basis, and strongly predict both the frequency of cannabis use and associated adverse outcomes.

**Methods:**

We employed a daily web survey over a 28-day period to assess daily motives and open-ended self-reported contexts of nonmedical cannabis use. Participants, aged 22–76 (*n* = 48, Mean = 48.8, SD = 17.1; 64 % female; 22 % African American), completed a baseline web survey on demographics and mental health and a follow-up web survey on mental health at present and cannabis use behaviors during the study. We applied latent transition analysis with random intercepts (RI-LTA) to analyze the data.

**Results:**

In our descriptive analyses, we identified four types of transitions in weekly latent motive classes, particularly transitions toward motives dominated by sleep aid and cannabis availability. Participants experiencing these transitions reported higher subsequent cannabis use frequency. Open-ended contextual information revealed behaviors such as cannabis use related to managing sleep disturbances, anxiety triggered by daily stressors, and recovery from medical treatments (e.g., chemotherapy).

**Conclusions:**

Our findings underscore the value of monitoring short-term transitions in cannabis use motives and contexts. This approach can inform the timing and content of adaptive interventions, proactively addressing the contexts and motives to prevent adverse cannabis use outcomes. Additional replications of this approach using larger samples are needed.

## 1. Introduction

### 1.1. Adverse health outcomes related to cannabis use

Given the rising prevalence of daily cannabis use in the United States, it is likely that more people will seek or need treatment for adverse consequences of frequent cannabis use (Caulkins, 2024; Choi et al., 2024; McCabe et al., 2021; Patrick et al., 2023). Indeed, the prevalence of young adults aged 19–30 reporting past-year cannabis use and daily cannabis use has recently reached its highest level ever, according to the Monitoring the Future (MTF) panel study (Patrick et al., 2023). Past-year use was reported by approximately 44 % of young adults in 2022, an increase from five years ago (35 % in 2017) and 10 years ago (28 % in 2012). Daily cannabis use also reached its highest level reported in 2022 (11 %), which was greater than five years (8 % in 2017) and 10 years ago (6 % in 2012) (Patrick et al., 2023).

Past-year cannabis use by adults 35–50 years old continued a long-term upward trajectory to reach all-time highs in 2022 (28 %). This had increased from the previous year (25 % in 2021) and five years ago (17 % in 2017), and more than doubled compared to 10 years ago (13 % in 2012) (Patrick et al., 2023). Between 1992 and 2022, there has been a 15-fold increase in the per capita rate of reporting daily or near daily cannabis use (Caulkins, 2024). Potential adverse consequences associated with daily cannabis use include development of cannabis use disorder (CUD), cannabis-related psychosis or psychiatric disorders, and impaired driving, among others (Di Forti et al., 2019; Lapham et al., 2023; Sultan et al., 2023). Although CUD treatment admissions may decline after the legalization of recreational cannabis use (Bass et al., 2024), among adults with past-year cannabis use, 30.3 % had CUD, and only 9.6 % received substance use treatment; those with severe CUD were more likely to receive substance use treatment (Choi et al., 2024).

### 1.2. Adaptive interventions as treatment strategies

Adaptive interventions (ADIs) are flexible treatment strategies that evolve over time to address the changing needs and behaviors of an individual and can lead to effective prevention and treatment, reducing cannabis use and associated consequences over time (Nahum-Shani et al., 2022; Nahum-Shani et al., 2012; Nahum-Shani et al., 2018; Perski et al., 2022). Recent studies tailored real-time messages supporting self-efficacy and prompting consideration of coping strategies for adolescents and young adult patients to reduce cannabis use according to self-reported momentary triggers for use, desire to use, or recent use; results showed that momentary cannabis use decreased over the course of the study, and that the odds of use were significantly lower in the intervention and follow-up phases than in the baseline phase (Shrier et al., 2014, 2018).

Lee et al. (2010) evaluated a brief web-based personalized feedback intervention for incoming college students who had recent cannabis use and suggested that the context of cannabis use may be influential during the first few months of college, and interventions prior to matriculation may have limited effect. In addition to prevention studies, Mason et al. (2025) tested a tailored text message-delivered CUD treatment with young adults and found indirect effects on reduction in cannabis use through readiness to change and protective behavioral strategies. Researchers compared several ADIs for the treatment of youth CUD and emphasized the need to explore effective tailoring strategies (Stanger et al., 2020). Developing effective ADIs requires collecting and analyzing relevant, time-varying auxiliary information, such as motives and contextual factors for cannabis use (Bonar et al., 2021, 2022, 2023).

### 1.3. Time-varying motives and implications of transitions in motives for adverse health outcomes

Several studies have found that certain types of motives for cannabis use, particularly coping and conformity, were linked to more problematic cannabis use behaviors and/or CUD (Arterberry et al., 2021; Bonar et al., 2017; Bresin and Mekawi, 2019; Cooper et al., 2016; Espinosa et al., 2023; Lee et al., 2007; Patrick, Schulenberg, O’Malley, Johnston, et al., 2011; Patrick et al., 2016; Patrick, Evans-Polce, et al., 2019; Patrick, Fairlie, et al., 2019). Previous studies typically measured motives as static traits during baseline data collection (Votaw and Witkiewitz, 2021). However, motives can vary within an individual over time (Bray et al., 2021; Graupensperger et al., 2021; Patrick, Schulenberg, O’Malley, Maggs, et al., 2011; Pearson et al., 2020; Votaw and Witkiewitz, 2021; West et al., 2024), and transitions in motives (i.e., changes in motives from one point in time to another) may predict cannabis use behaviors (e.g., frequency of use) at later points in time. For example, West et al. (2024) used latent transition analysis with random intercepts (RI-LTA) to associate transitions in motives with several distal outcomes of cannabis use and found that the higher the probabilities of specific types of transitions, the stronger the likelihood of greater cannabis use in the future. Thus, collecting and analyzing time-varying motives may help to inform ADIs, providing clinicians with actionable information on different motives (e.g., social vs. coping) or combinations thereof (Nahum-Shani et al., 2012, 2022).

### 1.4. Contextual factors for cannabis use

The measurement of motives, often referred to as reasons, for cannabis use dates back to the theoretical motivational model and its empirical support that informed the prevention and treatment of alcohol use (Cooper, 1994; Cox and Klinger, 1988, 1990, 2011). Cooper et al. (2016) reviewed the literature on motives for alcohol, cannabis, and tobacco use and substantiated the existence of similar types of motives for cannabis use, but it should be noted that researchers have also identified more detailed motive subscales for cannabis use (Blevins et al., 2016; Bohnert et al., 2018; Lee et al., 2009). While previous studies have primarily assessed motives for cannabis use as static traits, time-varying motives, as momentary processes in daily life, have stemmed from the interaction between contextual factors, such as the broader context, including historical factors (e.g., past cannabis use reinforcement) and traits (e.g., individual differences in impulsivity), as well as the immediate context, including internal states (e.g., negative affect, withdrawal, distress tolerance), situations (e.g., cannabis availability, day of week), and alternative incentives (e.g., coping skills, reinforcing activities) (Votaw and Witkiewitz, 2021). It is important to consider these contextual factors as well as the timing of interventions over time (Lee et al., 2010). However, no studies to date have assessed these contextual factors and the interactions among these factors in relation to motives and the health outcomes of cannabis use.

### 1.5. Research questions

Previously published secondary analyses of longitudinal data from four studies (West et al., 2024) have revealed associations between specific types of transition in motives and the likelihood of greater future cannabis use. The time-varying daily motives for cannabis use were assessed from established scales, and these data provided little information about the contexts in which transitions occurred, which would help clinicians to develop interventions. Furthermore, in three of those four studies, the samples were exclusively youth and young adults. The present study extends this previous assessment by collecting open-ended self-reported contexts of use in a small but socio-demographically diverse sample and analyzing transitions in motives and associated health outcomes. Our two primary research questions are as follows:

1. Are short-term transitions in time-varying motives for cannabis use associated with distal measures of cannabis use behaviors in a sample with a wide range of socio-demographic characteristics?
2. Are certain types of transitions associated with particular cannabis use contexts that could contribute to the development of ADIs?

## 2. Methods

### 2.1. Participants

The present study was approved by our University IRB (Study ID #HUM00202179). Participants were recruited from the Michigan Institute for Clinical & Health Research (MICHR) database website through an advertisement where prospective participants could either click a link, or click the “I Am Interested!” button, and we sent the link to the email address on file with MICHR. Prospective participants completed the screener survey on the web, and those who had used cannabis in the past 30 days were eligible to participate in the study. We set balanced quotas for five age groups (18–30, 31–50, 51–60, 61–70, and 70 +), sex, and ethnic minorities to ensure a diverse sample. Selected participants (*n* = 50) received web surveys by email and text (if a phone number was provided) and completed a baseline survey on demographics and mental health, 28 daily surveys on motives for cannabis use (if cannabis use was indicated yesterday), and a follow-up survey on mental health at present and a variety of cannabis use behaviors over the past month. Only one participant opted out after the baseline survey; for the 28-day survey, 49 participated at least 19 days, 48 at least eight days, and 47 at least one day; and all 49 participants completed the follow-up survey.

### 2.2. Measures

#### 2.2.1. Baseline survey on demographics and mental health

Eligible participants completed a baseline survey that assessed demographics and mental health. We assessed positive mental health using the Flourishing Scale (Diener et al., 2010), depression using the Patient Health Questionnaire (PHQ-9; Kroenke et al., 2001), anxiety using the Generalized Anxiety Disorder scale (GAD-7; Spitzer et al., 2006), and loneliness using the Three-Item Loneliness Scale (Hughes et al., 2004).

#### 2.2.2. Daily survey on motives for cannabis use

One week after the baseline survey invitation, participants received a survey once a day for 28 consecutive days and were asked to complete it each day. We asked participants how many times they had used cannabis on the previous day, separated by at least 1 h in between each use. If they had used at least once, they were asked the motives behind that use. We assessed ten motives that were modified from the Comprehensive Marijuana Motives Questionnaire (Lee et al., 2009) and included a pain relief motive (Brammer et al., 2022). These motives had high levels of endorsement and tended to change over time, drawing on the results of previous secondary analyses (West et al., 2024). We asked participants to indicate whether or not (yes/no) they used cannabis yesterday to enjoy the feeling or get high, to avoid feeling left out, to expand awareness or understand things differently, to relax or improve mood, to have fun or make a social gathering more fun, to feel more self-confident or more creative, to help you sleep, because it is a low-risk substance, because it was readily available, or to help relieve physical pain, and we asked them to explain other reasons, if any. Participants could endorse multiple motives within a single day. To assess the contextual factors, we also asked participants to describe what they were doing when they used cannabis in an open-ended text box. Once each day, a reminder text and e-mail were both sent to non-responsive participants.

#### 2.2.3. Follow-up survey on mental health at present and cannabis use behaviors during the study

The day after the last daily survey was sent, participants received the follow-up survey on mental health at present and cannabis use behaviors during the study. We used the same mental health scales as in the baseline survey. Regarding cannabis use behaviors, we asked participants how many occasions they had used cannabis in the past week.

### 2.3. Data analysis

All analyses were conducted using R version 4.3.3 (R Core Team, 2023) and M*plus* version 8.9 (Muthen and Muthen, 2017). The code is available in the supplemental materials. We applied RI-LTA (Muthén and Asparouhov, 2022) in M*plus* to the daily indicators of motives for cannabis use and cannabis use behaviors collected in the study. We found that day-to-day transitions in latent motive classes were rare, so we recoded the daily indicators of motives as weekly indicators that represented whether any particular motive was endorsed on weekdays (i.e., Monday through Thursday) and/or the weekend (i.e., Friday through Sunday) in a given week. For example, if a participant endorsed a given motive on any weekday of the week, then the corresponding weekly indicator for that motive became activated.

We estimated the probabilities of membership in latent classes characterized by specific motives in Week 1 and Week 4 in the RI-LTA, which separates between-subject variation from the within-subject latent class transitions over time by allowing random intercept variation (Muthén and Asparouhov, 2022). We also estimated the probabilities of transitioning from one motive class to another between Week 1 and Week 4 as well as the corresponding means of the past-week cannabis use occasions in the follow-up survey as the distal measure of future cannabis use behavior. We excluded indicators for Week 2 and Week 3 from the analysis due to the limited sample size relative to the number of parameters that would need to be estimated if these two intermediate weeks were included.

As demonstrated by West et al. (2024), we used commonly reported measures of model fit for RI-LTA (e.g., BIC and entropy) to ultimately select the best-fitting models in terms of the number of latent motive classes at the two time points. We then included the distal outcome to estimate the transition-specific means. It should be noted that when following this manual two-step approach, the inclusion of the distal outcome in the second step could influence the latent class formation, and the transition-specific distribution of the distal outcome cannot be interpreted as the direct effect of a transition, as the distal outcome itself was also considered an indicator of the latent motive class variable (Nylund-Gibson et al., 2019). In these cases, it is indeed recommended to use multi-step methods such as the BCH method to ensure that the latent class membership does not change, as the reviewer notes (Asparouhov and Muthén, 2021). We re-ran all of our analyses using the BCH method for RI-LTA (Muthén and Asparouhov, 2025), which had not been fully developed when we first conducted the analyses for this paper, but our small sample size in the present study unfortunately led to model non-convergence (see the supplemental material for syntax). Although it is assumed that the measurement of the latent classes is the same across time in most applications of LTA, the measurement of the latent motive classes can be qualitatively different across time, and the transition probabilities are interpreted as the probability of changing between qualitatively distinct motive classes (Nylund-Gibson et al., 2023).

## 3. Results

### 3.1. Demographics

Of the 50 participants, two did not report the past-week cannabis use occasions in the follow-up survey and were excluded from the analysis. Table 1 shows the demographics and descriptive statistics.

### 3.2. Latent classes, transitions, and associated distal cannabis use behavior

We identified four types of transitions from two latent motive classes on weekdays in Week 1 to two classes on weekdays in Week 4, and estimated the associated means of the past-week cannabis use occasions in the follow-up survey. We excluded one motive assessing conformity (i.e., avoid feeling left out), for which the low probability of endorsement did not distinguish between motive classes. The patterns of transitions in the weekend latent motive classes were not evident, with most participants remaining in the same classes. We thus reported and interpreted the transitions in the weekday latent motive classes. See the supplementary materials for additional results related to the weekend latent classes and transitions.

**Fig. 1.**
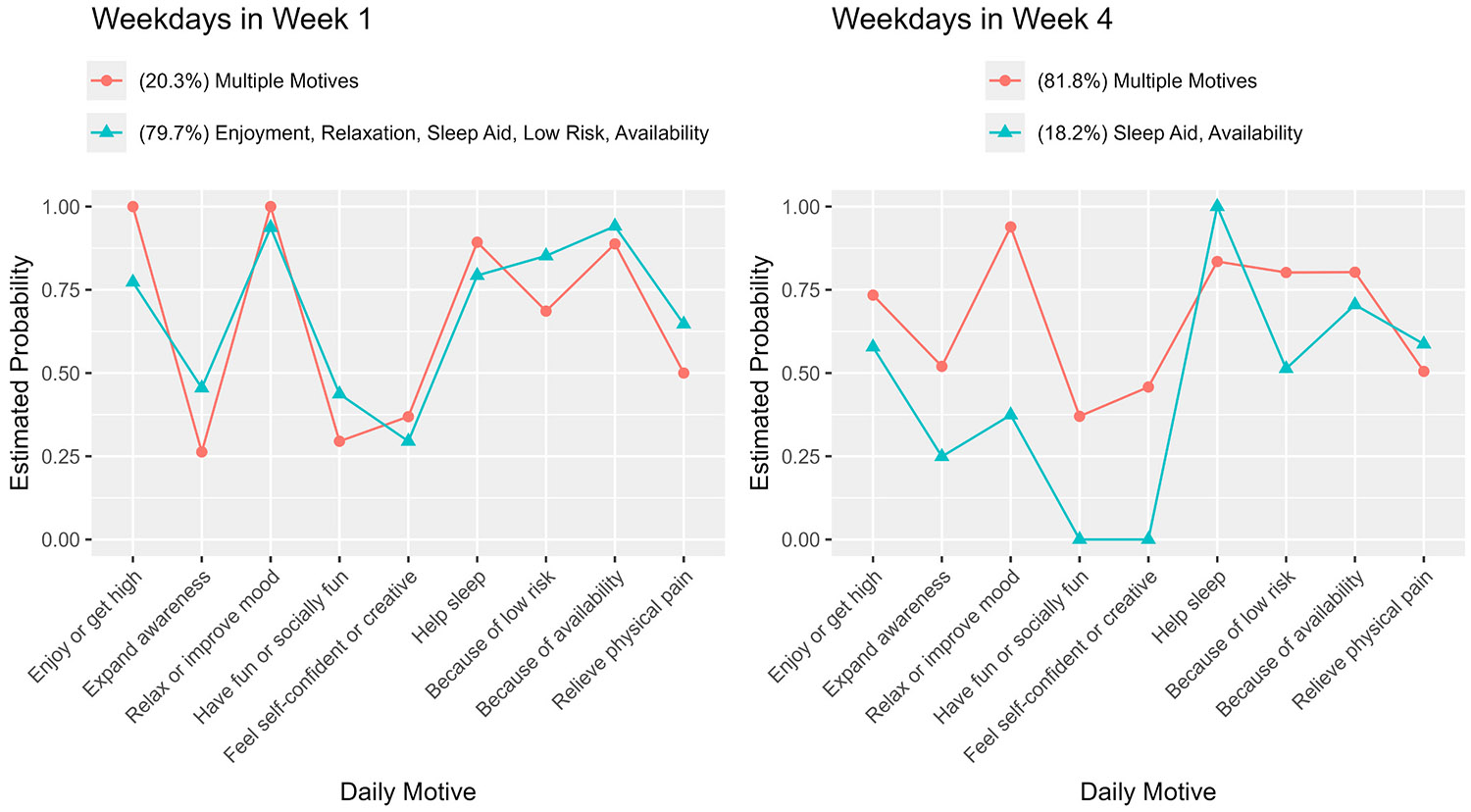
Estimated probabilities of endorsing motives in two classes on weekdays (Monday–Thursday) in Week 1 and Week 4 in the RI-LTA (*n* = 48; BIC = 1078.23; Entropy = 0.983). The percentage of the sample in each class is indicated in parentheses.

Fig. 1 shows the estimated probabilities of endorsing the various motives in two classes on weekdays in Week 1 and Week 4. In Week 1, one motive class was defined by multiple motives except for feeling more self-confident or more creative; another was primarily defined by enjoyment, relaxation, sleep aid, low risk, and availability; the separation of the two classes was moderate. In Week 4, compared with the multiple motives class in Week 1, the multiple motives class had relatively higher probabilities of endorsing cannabis use to feel more self-confident or more creative; the other motive class was primarily defined by sleep aid and availability.

While the cannabis use occasions asked about in the follow-up survey covered the past week that overlapped with Week 4 weekdays, these occasions also covered the Week 4 weekend, which was considered distal relative to the weekdays. Table 2 shows the estimated probabilities of the four types of transition and the estimated means of the follow-up past-week cannabis use occasions associated with each type of transition. From Week 1 to Week 4, transitioning from the motive class defined by enjoyment, relaxation, sleep aid, low risk, and availability to the motive class defined by sleep aid and availability (**Transition 4**) was associated with highest estimated mean of cannabis use occasions (45.00), although this transition was unlikely to happen (estimated probability of 0.05); remaining in the multiple motives class (**Transition 1**) or transitioning to the motive class defined by sleep aid and availability (**Transition 2**) was also associated with a high estimated mean of cannabis use occasions (16.00 and 12.36, respectively). In contrast, transitioning from the motive class defined by enjoyment, relaxation, sleep aid, low risk, and availability to the multiple motives class (**Transition 3**) was extremely likely to happen (estimated probability of 0.95) and was associated with the lowest estimated mean of cannabis use occasions (3.86). See the supplementary materials for demographic statistics and mental health assessments at baseline and follow-up for each type of transition.

Of the three transitions associated with high estimated means of follow-up past-week cannabis use occasions, only three participants experienced Transition 1, seven participants experienced Transition 2, and only two participants experienced Transition 4. We focused our qualitative analysis on participants going through Transitions 1, 2, and 4, which were associated with relatively high estimated means of cannabis use occasions.

### 3.3. Mental health in transitions 1, 2 and 4

Table 3, Table 4, and Table 5 focus on the mental health for each participant who experienced Transition 1, Transition 2, and Transition 4.

**Participant 1** shifted from mild to minimal depression symptoms. **Participant 2** shifted from feeling lonely to not feeling lonely. **Participant 3** shifted from mild to moderate depression symptoms, and from moderate to mild anxiety symptoms.

**Participant 4** shifted from minimal to mild depression symptoms, from minimal to moderate anxiety symptoms, and from not feeling lonely to feeling lonely. **Participant 8** shifted from minimal to mild depression symptoms, from mild to moderate anxiety symptoms, and from not feeling lonely to feeling lonely. **Participant 9** shifted from minimal to mild depression symptoms. Compared to these participants, the mental health of the other four participants shifted toward positive, especially **Participant 7**, who shifted from mild to minimal depression symptoms, and **Participant 10**, who shifted from moderate to minimal depression symptoms, from severe to minimal anxiety symptoms, and from feeling lonely to not feeling lonely.

**Participant 11** shifted from feeling lonely to not feeling lonely. **Participant 12** shifted from moderate to mild depression symptoms.

### 3.4. Other contextual factors for cannabis use in transitions 1, 2 and 4

Table 6, Table 7, and Table 8 show the self-reported contexts of cannabis use for each participant who experienced Transition 1, Transition 2, and Transition 4.

**Participant 3** provided details about the context of cannabis use. One particular incident mentioned was that on Tuesday during Week 1: “*Attended a social function for my partner’s birthday, celebrated at a number of different locations throughout the evening*”, and on Monday during Week 4: “*Used after a fight with partner to relax and help refocus myself, then at bedtime for relaxing and sleep*”. This seemed to be associated with the shift toward moderate depression symptoms. Relaxation and sleep aid motives also became more prominent in Week 4 compared to Week 1, while enjoyment and social fun motives were lacking.

**Participant 4** reported using cannabis on Thursday in Week 1 to “*aid with chemo-induced neuropathy*”. During Week 4, the participant used cannabis to cope with anxiety, specifically on Tuesday for mood stabilization, and on Thursday “*to help get through a very difficult day.*” This might be associated with the shift toward mild depression symptoms, moderate anxiety symptoms, and feeling lonely, while the participant did not mention these motives during Week 1. Although the participant did not provide more details in Week 4, the emphasis on these mental health-related motives suggested that their impact on cannabis use could have gone beyond the relaxation motive that was frequently mentioned during both weeks.

**Participant 8** provided some details during Week 1 but not in Week 4. In Week 1, the participant “*wanted to enjoy some lone time*”, “*was happy*”, and “*got stuck on problem, used it to enlighten myself and eventually pulled through*”. However, the participant felt lonely, more anxious and depressed in Week 4 and used more cannabis. It was hard to figure out potential contextual or other precipitating factors, because the only thing we knew was that the participant watched anime on Tuesday in Week 4. **Participant 9** did not provide too many details either. The shift toward mild depression symptoms might be associated with work that was mentioned on Tuesday and Wednesday in Week 4 but not in Week 1.

**Participant 12** reported using cannabis while planning activities, working outside, and talking with friends on the phone or in person during Week 1, specifically mentioning that it helped to focus on lots of tasks. However, during Week 4, the participant spent most of the time at home doing “*low energy house stuff*” and “*napping*”. Although it did not appear that the mental health of the participant was turning negative, on Wednesday and Thursday in Week 4, the participant experienced tiredness and headaches due to delayed healing from postchemotherapy complications, as well as feeling low and “*jittery from steroid drugs*”.

## 4. Discussion

The present study performed descriptive analyses of short-term transitions in time-varying motives for cannabis use and associated distal health outcomes in a small but socio-demographically diverse sample. We identified two latent classes of cannabis use motives on weekdays in Week 1—the multiple motives class and the motive class defined by enjoyment, relaxation, sleep aid, low risk, and availability-—and two on weekdays in Week 4—the multiple motives class and the motive class defined by sleep aid and availability. We found that three types of transitions were associated with frequent past-week cannabis use as assessed in the follow-up survey—remaining in the multiple motives class (**Transition 1**), transitioning from the multiple motives class in Week 1 to the sleep aid and availability class in Week 4 (**Transition 2**), and transitioning from the enjoyment, relaxation, sleep aid, low risk, and availability class in Week 1 to the sleep aid and availability class in Week 4 (**Transition 4**). However, these associations should not be interpreted causally.

Previous studies have suggested that remaining in or transitioning to the multiple motives class from one where fewer motives were endorsed generally had more frequent cannabis use in the future (Espinosa et al., 2023). Our analyses of Transition 1 also suggested that remaining in the multiple motives class was associated with frequent cannabis use. However, in our analyses of Transition 3, which was experienced by the majority of participants, transitioning to the multiple motives class was not associated with frequent cannabis use. Even though the endorsement of multiple motives was associated with adverse health outcomes, it may be impractical for clinicians to implement interventions that target all motives for cannabis use, and certain motives may be more influential. Bonn-Miller et al. (2014) and Drazdowski et al. (2021) found that the sleep aid motive was associated with increased and problematic cannabis use. Our analyses of Transition 2 and Transition 4 also suggested that specific motives such as sleep aid and availability appeared to have a greater impact on participants who experienced these transitions. These contradictory findings underscored the importance of understanding the context of cannabis use before acting to address the motives for its use.

To understand these transitions and associated cannabis use behaviors, we examined mental health as assessed in the baseline and followup surveys before and after transitions, as well as open-ended, self-reported contexts of cannabis use in the daily surveys. While depression, anxiety, and loneliness did not appear to be potential factors in cannabis use behavior for participants who experienced Transition 1 and Transition 4, we found that some participants who experienced Transition 2 experienced a shift to more severe depression symptoms, anxiety symptoms, and loneliness, which might have been associated with more frequent cannabis use. While we were working with a small sample given the intensive data collection protocol, this qualitative analysis provided interesting insights into the unique context associated with these types of transitions.

What types of ADIs might these results suggest? These interventions might focus on behavioral activation strategies to address depressive symptoms, anxiety management techniques, and exploring healthier alternatives for managing sleep disturbances. The contextual data collected from participants (e.g., stressors related to chemotherapy-induced neuropathy or acute anxiety triggered by specific daily challenges) further highlighted the need for context-sensitive interventions. Real-time interventions could proactively address identified contexts by delivering targeted support precisely when these contextual triggers arise. In current survey design, participants were instructed not to report a second use episode if it occurred within one hour of a previous episode, which may have reduced the amount of contextual information captured—particularly among individuals who segment a single smoking session into multiple parts or who consume edibles with prolonged effects. This constraint likely produced conservative estimates of contextual variability and should be relaxed or redesigned in future studies.

As Lee et al. (2010) emphasized in an intervention study targeting the transitional period before and after matriculation into college, the timing of interventions may have important implications, as the context of cannabis use can be influential during the first few months of college. In the present study, we detected transitional periods with the RI-LTA and attempted to understand these transitions from self-reported contexts that indicated timing of intervention. Addressing contextual factors of cannabis use may facilitate readiness to change and protective behavioral strategies that mediate substance use treatment effects, such as text-delivered brief motivational interviewing-informed treatment (Mason et al., 2025). Although the sample size was small in the present study, we see this as a promising approach for combining statistical analysis and qualitative assessment in the development of ADIs. Future research could replicate this approach with larger samples and encourage participants to provide detailed contextual information on cannabis use. With larger samples, clinicians may be able to associate particular cannabis use contexts with certain types of transitions and develop tailored interventions and treatment strategies accordingly.

### 4.1. Limitations

The present study employed self-administered web surveys that allowed participants to disclose more information about the contexts of cannabis use relative to prior studies (e.g., Williams et al., 2022). In this way, we were able to conduct more frequent or regular assessments at lower costs. However, several limitations should be considered.

First, the sample size restricted the generalizability of the findings and limited statistical power. The quota-based sampling approach limited our ability to generalize the findings to broader populations. We intended to demonstrate the feasibility of this approach in a sample that included representatives from all groups, including adults. The results reported in the present study should be treated as descriptive and specific to this small pilot study, and could be used to motivate testable hypotheses for future intervention studies. Future research should replicate the present study with larger, more representative samples that are reflective of cannabis use distributions across various age groups, ultimately producing more generalizable and informative findings that can robustly inform the designs of future interventions. Larger samples would also facilitate the identification of more potential motive classes with clear distinctions, as found in a previous study (West et al., 2024), and allow for the use of multi-step methods such as the BCH method to avoid the undesirable shift of motive class membership (Asparouhov and Muthén, 2021), the analyses of transitions between more than two time points, and the inclusion of confounders such as baseline cannabis use frequency.

Second, additional contextual variables, such as religious affiliation, ethnicity, disability status, veteran status, or social acceptance of cannabis use within the community were not assessed in the present study. These variables may impact motives and contexts of cannabis use, suggesting that future studies should incorporate these factors for a more comprehensive understanding. In addition, the present study did not distinguish between medical and non-medical cannabis use cases. Exploring behavioral differences between these contextual groups could provide valuable insights for tailoring ADIs; for example, given the changes that participants may experience, the ADI may require a revision in response to changes in the motive class throughout the observation period.

Finally, although the present study implemented daily web surveys, the retrospective self-reporting of cannabis use motives and contexts may be subject to memory bias, potentially impacting data accuracy. Future research could employ ecological momentary assessment (EMA) techniques to minimize recall bias. Also, because motives were collected once per day, our ability to characterize within-day context was constrained when multiple use episodes occurred in close succession. This may have reduced contextual granularity—particularly among participants who segment a single smoking session into multiple parts or who consume edibles with prolonged effects—yielding conservative estimates of contextual variability. Future work should relax this constraint and use EMA or event-based, time-stamped logging to capture closely spaced, overlapping, or extended episodes so that intervention timing more accurately reflects lived patterns of use.

The present study drew on a previous study in which motives were assessed at different frequencies (West et al., 2024) to select measures for daily motives. Future research should validate the measures and ensure that the measures have favorable psychometric properties and measurement invariance across time in daily surveys.

In conclusion, the approach demonstrated in the present study can be expanded to identify and understand transitional periods in motives for cannabis use that are associated with emerging mental health symptoms and problematic cannabis use in the future. The open-ended self-reported contextual information could be used to further inform the design of ADIs that effectively address active symptoms and curb problematic cannabis use over time, ideally leading to improvements in health.

## Supplementary Material

Supplemental Materials

## Figures and Tables

**Table 1:** Descriptive statistics (*n* = 48).

	Mean	SD
Age	48.79	17.06
	*n*	%
Sex		
Female	32	64
Male	18	36
Sexual Orientation		
Heterosexual	42	84
Other	8	16
Race		
African American	11	22
White	31	62
Other	8	16
Citizenship		
US Citizen	48	96
Other	2	4
Education Level		
8th Grade or Lower	1	2
High School Degree	11	22
Some College (But No College Degree)	8	16
Associate’s Degree	4	8
Bachelor’s Degree	15	30
Graduate Degree	11	22

**Table 2:** Estimated transition probabilities and estimated means of cannabis use occasions in the past week in the follow-up survey (*n* = 48).

	Week 1 Weekdays Motive Class	Week 4 Weekdays Motive Class	Estimated Transition Probability	Estimated Mean of Follow-up Past-Week Cannabis Use Occasions (SE)
Transition 1	Multiple Motives	Multiple Motives	0.31	16.00 (1.67)
Transition 2	Multiple Motives	Sleep Aid, Availability	0.69	12.36 (3.66)
Transition 3	Enjoyment, Relaxation, Sleep Aid, Low Risk, Availability	Multiple Motives	0.95	3.86 (0.53)
Transition 4	Enjoyment, Relaxation, Sleep Aid, Low Risk, Availability	Sleep Aid, Availability	0.05	45.00 (2.12)

**Table 3:** Mental health at baseline and follow-up for each participant who experienced Transition 1.

	Participant 1	Participant 2	Participant 3
Baseline			
FS	56	49	46
PHQ-9	Mild	Mild	Mild
GAD-7	Minimal	Mild	Moderate
UCLA-3	Not lonely	Lonely	Not lonely
Follow-up			
FS	56	48	48
PHQ-9	Minimal	Mild	Moderate
GAD-7	Minimal	Mild	Mild
UCLA-3	Not lonely	Not lonely	Not lonely
Follow-up Past-Week Cannabis Use Occasions	14	14	20

Note: FS—Flourishing Scale for positive mental health; PHQ-9—Patient Health Questionnaire for depression; GAD-7—Generalized Anxiety Disorder for anxiety; UCLA-3—Three-Item Loneliness Scale for loneliness.

**Table 4:** Mental health at baseline and follow-up for each participant who experienced Transition 2.

	Participant 4	Participant 5	Participant 6	Participant 7	Participant 8	Participant 9	Participant 10
Baseline							
FS	50	49	48	36	44	41	35
PHQ-9	Minimal	Minimal	Minimal	Mild	Minimal	Minimal	Moderate
GAD-7	Minimal	Minimal	Minimal	Mild	Mild	Minimal	Severe
UCLA-3	Not lonely	Not lonely	Not lonely	Lonely	Not lonely	Not lonely	Lonely
Follow-up							
FS	51	52	50	51	45	44	53
PHQ-9	Mild	Minimal	Minimal	Minimal	Mild	Mild	Minimal
GAD-7	Moderate	Minimal	Minimal	Mild	Moderate	Minimal	Minimal
UCLA-3	Lonely	Not lonely	Not lonely	Lonely	Lonely	Not lonely	Not lonely
Follow-up Past-Week Cannabis Use Occasions	18	15	7	10	13	10	12

**Table 5:** Mental health at baseline and follow-up for each participant who experienced Transition 4.

	Participant 11	Participant 12
Baseline		
FS	44	43
PHQ-9	Minimal	Moderate
GAD-7	Minimal	Mild
UCLA-3	Lonely	Not lonely
Follow-up		
FS	45	44
PHQ-9	Minimal	Mild
GAD-7	Minimal	Mild
UCLA-3	Not lonely	Not lonely
Follow-up Past-Week Cannabis Use Occasions	48	42

**Table 6:** Context of cannabis use for each participant who experienced Transition 1.

Participant 1		Participant 2	Participant 3
Week 1 Monday	I was camping. spending with family & friends	Anxiety; Cleaning house	Was camping which included a number of different activities such as fishing, hiking, etc.
Week 1 Tuesday	Every day chores	Anxiety; watching tv	Attended a social function for my partners birthday, celebrated at a number of different locations throughout the evening
Week 1 Wednesday	Every day activities	Anxiety; Sitting outside relaxing	Went out with a few friends to celebrate a birthday and partook then rather than drinking, then before bed for sleeping
Week 1 Thursday	Completing paperwork for family, phone calling for aoots, household chores, watching television	Anxiety and Depression; Watching tv	Used after work to relax while doing house chores and then before bed for sleep
Week 4 Monday	Relaxing, chores, watch tv, crosswords	Grieving; Nothing	Used after a fight with partner to relax and help refocus myself, then at bedtime for relaxing and sleep
Week 4 Tuesday	Life, sewing, traveling, watching movies, paperwork		Used in the evening to unwind and relax after work then before bed for sleep
Week 4 Wednesday	Crafting, cleaning hime, relaxing, gaming	Watching tv	Used several times after work while doing house chores then before bed for relaxing and sleep
Week 4 Thursday	Household chores, paperwork, reading, watching tv	Depression; Nothing	Used while cleaning car and doing other miscellaneous tasks around house, then some while watching tv before bed to sleep
Follow-up Past-Week Cannabis Use Occasions	14	14	20

**Table 7:** Context of cannabis use for each participant who experienced Transition 2.

	Participant 4	Participant 5	Participant 6	Participant 7	Participant 8	Participant 9	Participant 10
Week 1 Monday	Pain relief while doing everyday activities	Walking around my yard to observe my plants and garden.	Relaxing at home before bedtime.	I used it when I woke up and then periodically throughout the day	Was home didn’t go out wanted to enjoy some lone time and I was listening to music.	Watching tv. Socializing	Relaxing in my chair, listening to music
Week 1 Tuesday	Relaxing	Sitting in my living room watching TV. Walking around in my yard.	Relaxing at home with the family	making breakfast, driving, working, after dinner with friends and before bed	Was relaxing after a day’s work	Running errands, cooking	Relaxing
Week 1 Wednesday	exercising, relaxing	Sitting in my living room	Relaxing at home.	after dinner and right before bed	I was happy	Cleaning, socializing	Watching a movie
Week 1 Thursday	Aid with chemo-induced neuropathy; Relaxing. Taking a break. Performing chores.	Appetite enhancing; Sitting in my living room.	Having dinner with family.	before bed	Got stuck on problem, used it to enlighten myself and eventually pulled through	Social media. Socializing with a friend	I was listening to music
Week 4 Monday	Anxiety; Chores, relaxing		Getting ready to go to bed.			Playing with grandkids	Relaxing
Week 4 Tuesday	Anxiety, depression; PT Exercise, mood stabilizer	While watching TV before bed	Getting ready for bed.	before bed	Watching anime	Looking for work	Relaxing
Week 4 Wednesday	Anxiety, depression; Relaxing, chores	Sitting in my living room watching TV.	Relaxing before bed.			Working	Relax
Week 4 Thursday	To help get through a very difficult day; Anxiety, coping	Sitting on my couch while watching TV	Relaxing before bedtime.			Watching tv	Relaxing
Follow-up Past-Week Cannabis Use Occasions	18	15	7	10	13	10	12

**Table 8:** Context of cannabis use for each participant who experienced Transition 4.

	Participant 11	Participant 12
Week 1 Monday	I sit in my porch or in my garage and smoke about a half joint at a time.	Cleaning house, making food, watching tv
Week 1 Tuesday	Sitting on the porch, i don’t smoke in the house, either getting ready for bed, waking up, or because i felt like a smoke.	Taking a couple puffs off a pipe seems to quiet the endless loop of chatter reminding me of all the things that need my attention. Often helps me focus on tasks. Can also demotivate further action if tired; Planning the day. Working outside. Talking with friends on phone or in person
Week 1 Wednesday	Sitting on my porch each time getting ready for bed or waking up or just relaxing and watching tv	Lots of tasks to accomplish; Establishing plan of action/activities. Stayed away from morning news.
Week 1 Thursday	Sitting on the porch as usual	Used prior to appointment in morning and after returning home in evening
Week 4 Monday	Waking up, going to bed, decorating, sitting on the porch	Hanging at home
Week 4 Tuesday	Waking up, going to bed, doing projects, sitting on the porch	Home, few errands
Week 4 Wednesday	Waking up, going to bed, playing video games, sitting on the porch	Post chemo after 5 week delay from complications healing; Laying low, jittery from steroid drugs
Week 4 Thursday	Waking up, going to sleep, playing video games	Laying low, post chemo, a bit tired and headachey; Low energy house stuff, napping
Follow-up Past-Week Cannabis Use Occasions	48	42

## Data Availability

The data used in this research are not currently available. Steps are actively being taken to make the data available through openICPSR.

## Appendix A. Supporting information

Supplementary data associated with this article can be found in the online version at doi:10.1016/j.drugalcdep.2025.112974
