# Supplementary material for: Daily web survey data collection of time-varying cannabis use motives and contexts, with implications for adaptive interventions: A pilot study
Source: Drug Alcohol Depend. Author manuscript; Available in PMC 2026 Apr 29. (PMC13126555; doi:10.1016/j.drugalcdep.2025.112974)
Supplement: Supplemental Materials [file NIHMS2164999-supplement-Supplemental_Materials.docx]

Daily Web Survey Data Collection of Time-Varying Cannabis Use Motives and Contexts, with Implications for Adaptive Interventions: A Pilot Study

# Supplemental Materials

**Table A1.** Descriptive statistics of demographics by transitions.

|  | Transition 1  *n* = 3  M (SD) | Transition 2  *n* = 7  M (SD) | Transition 3  *n* = 36  M (SD) | Transition 4  *n* = 2  M (SD) |
| --- | --- | --- | --- | --- |
| Age | 50.67 (22.37) | 53.14 (17.28) | 47.97 (17.12) | 54.00 (18.38) |
|  | *n* | *n* | *n* | *n* |
| Sex |  |  |  |  |
| Female | 3 | 4 | 23 | 1 |
| Male | 0 | 3 | 13 | 1 |
| Sexual Orientation |  |  |  |  |
| Heterosexual | 3 | 6 | 29 | 2 |
| Other | 0 | 1 | 7 | 0 |
| Race |  |  |  |  |
| African American | 0 | 2 | 9 | 0 |
| White | 2 | 3 | 23 | 2 |
| Other | 1 | 2 | 4 | 0 |
| Citizenship |  |  |  |  |
| US Citizen | 3 | 7 | 34 | 2 |
| Other | 0 | 0 | 2 | 0 |
| Education Level |  |  |  |  |
| 8^th^ Grade or Lower | 0 | 0 | 1 | 0 |
| High School Degree | 1 | 3 | 6 | 1 |
| Some College (But No College Degree) | 1 | 1 | 6 | 0 |
| Associate’s Degree | 0 | 2 | 2 | 0 |
| Bachelor’s Degree | 0 | 0 | 13 | 1 |
| Graduate Degree | 1 | 1 | 8 | 0 |

**Table A2.** Mental health at baseline and follow-up by type of transition.

|  | Transition 1  *n* = 3 | | Transition 2  *n* = 7 | | Transition 3  *n* = 36 | | Transition 4  *n* = 2 | |
| --- | --- | --- | --- | --- | --- | --- | --- | --- |
|  | Baseline | Follow-up | Baseline | Follow-up | Baseline | Follow-up | Baseline | Follow-up |
|  | M (SD) | | M (SD) | | M (SD) | | M (SD) | |
| FS | 50.33 (5.13) | 50.67 (4.62) | 43.29 (6.16) | 49.43 (3.51) | 43.06 (9.57) | 43.94 (11.96) | 43.50 (0.71) | 44.50 (0.71) |
|  | *n* (%) | | *n* (%) | | *n* (%) | | *n* (%) | |
| PHQ-9 |  |  |  |  |  |  |  |  |
| Minimal |  | 1 (33.4) | 5 (71.4) | 4 (57.1) | 11 (32.4) | 16 (44.5) | 1 (50.0) | 1 (50.0) |
| Mild | 3 (100.0) | 1 (33.3) | 1 (14.3) | 3 (42.9) | 12 (35.3) | 12 (33.3) |  | 1 (50.0) |
| Moderate |  | 1 (33.3) | 1 (14.3) |  | 10 (29.4) | 4 (11.1) | 1 (50.0) |  |
| Moderately Severe |  |  |  |  | 1 (2.9) | 4 (11.1) |  |  |
| GAD-7 |  |  |  |  |  |  |  |  |
| Minimal | 1 (33.4) | 1 (33.3) | 4 (57.1) | 4 (57.1) | 20 (57.1) | 21 (58.3) | 1 (50.0) | 1 (50.0) |
| Mild | 1 (33.3) | 2 (66.7) | 2 (28.6) | 1 (14.3) | 6 (17.1) | 9 (25.0) | 1 (50.0) | 1 (50.0) |
| Moderate | 1 (33.3) |  |  | 2 (28.6) | 8 (22.9) | 4 (11.1) |  |  |
| Severe |  |  | 1 (14.3) |  | 1 (2.9) | 2 (5.6) |  |  |
| UCLA-3 |  |  |  |  |  |  |  |  |
| Not lonely | 2 (66.7) | 3 (100.0) | 5 (71.4) | 4 (57.1) | 19 (52.8) | 22 (61.1) | 1 (50.0) | 2 (100.0) |
| Lonely | 1 (33.3) |  | 2 (28.6) | 3 (42.9) | 17 (47.2) | 14 (38.9) | 1 (50.0) |  |
| Note: FS—Flourishing Scale for positive mental health; PHQ-9—Patient Health Questionnaire for depression; GAD-7—Generalized Anxiety Disorder for anxiety; UCLA-3—Three-Item Loneliness Scale for loneliness. | | | | | | | | |

**Table A3.** Estimated transition probabilities and estimated means of cannabis use occasions in the past week in the follow-up survey (*n* = 48).

|  | Week 1 Weekend Motive Class | Week 4 Weekend Motive Class | Estimated Transition Probability | Estimated Mean of Follow-up Past-Week Cannabis Use Occasions (SE) |
| --- | --- | --- | --- | --- |
| Transition 1 | Enjoyment, Relaxation, Availability | Relaxation, Availability | 0.94 | 5.31 (0.85) |
| Transition 2 | Enjoyment, Relaxation, Availability | Multiple Motives | 0.06 | 45.00 (2.12) |
| Transition 3 | Multiple Motives | Relaxation, Availability | 0.17 | 4.32 (0.40) |
| Transition 4 | Multiple Motives | Multiple Motives | 0.83 | 7.79 (1.41) |


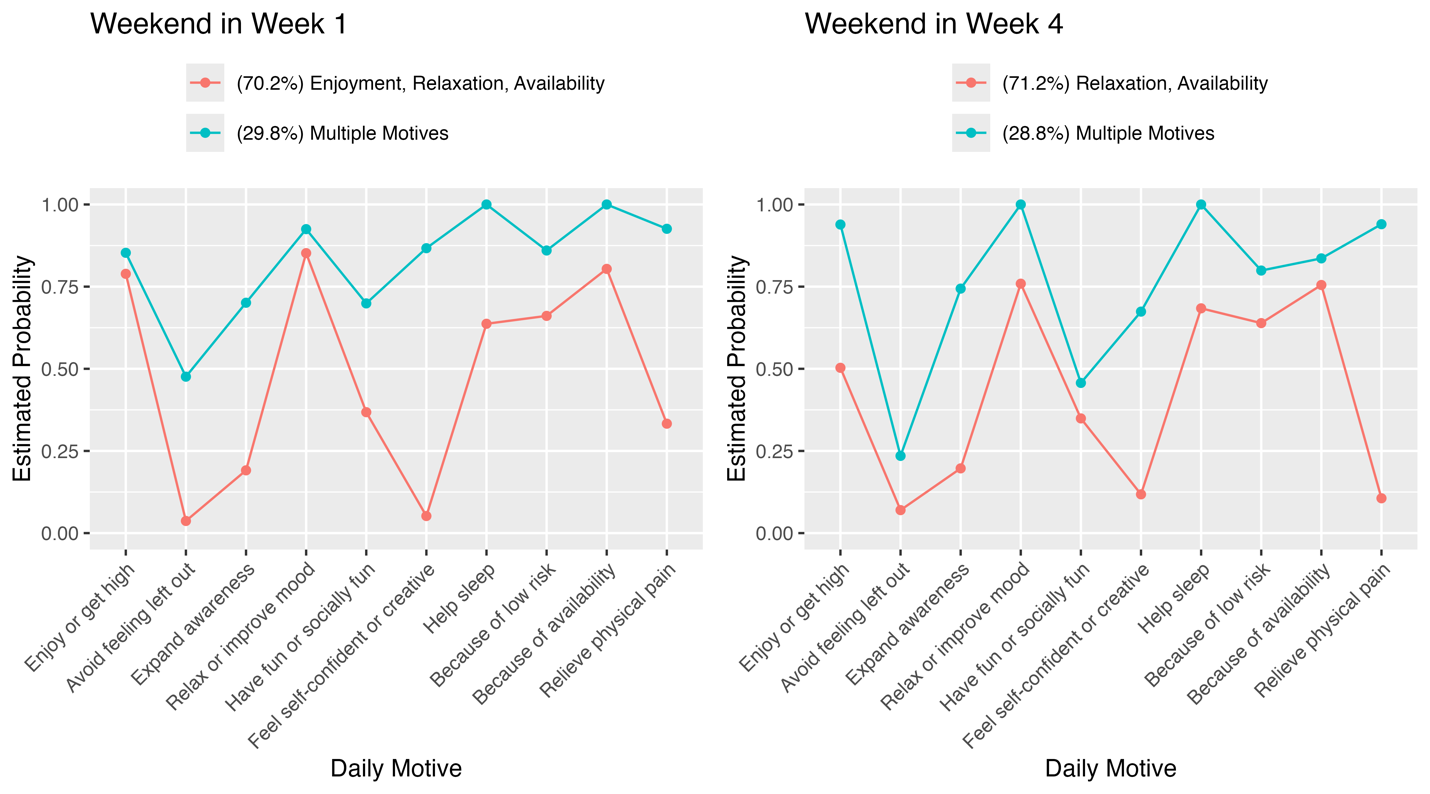


**Figure A.** Estimated probabilities of endorsing motives in two classes on weekend (Friday–Sunday) in Week 1 and Week 4 in the RI-LTA (*n* = 48; BIC = 1321.33; Entropy = 0.953). The percentage of the sample in each class is indicated in parentheses.
